# SUMO in the DNA damage response

**DOI:** 10.18632/oncotarget.4605

**Published:** 2015-06-23

**Authors:** Ivo A. Hendriks, Alfred C.O. Vertegaal

**Affiliations:** Department of Molecular Cell Biology, Leiden University Medical Center, Leiden, the Netherlands

**Keywords:** Chromosome Section, SUMOylation, ubiquitin, methylation, DNA damage response, DNA repair

Post-translational modification by Small Ubiquitin-like Modifiers (SUMOs) is critical for all eukaryotic life [1]. SUMO, like ubiquitin, is conjugated to lysine residues in target proteins through an enzymatic cascade involving E1, E2 and E3 enzymes, and SUMOylation of proteins can be reversed through the action of SUMO-specific proteases.

SUMO is generally known as a modifier of transcription and is a key player in the DNA damage response (DDR), with various DNA repair factors being SUMOylated in response to DNA damage, and an overall increased presence of SUMOylation at sites of DNA damage [2].

SUMO non-covalently interacts with other proteins through SUMO interaction motifs (SIMs). A well-known protein containing SIMs is the E3 ubiquitin ligase RNF4, which functions as a SUMO-targeted ubiquitin ligase. RNF4 generally ubiquitylates polySUMOylated proteins in order to facilitate their degradation, and performs an important role in the DDR [3]. Through multiple interactions between SUMOs and SIMs within different subunits of protein complexes, SUMO/SIM interactions are believed to serve as a mechanism to stabilize macromolecular structures [4].

Recently, major advances in proteomics have led to a more in-depth characterization of the SUMOylated proteome, uncovering over a thousand SUMO substrates and many thousands of SUMOylated lysines [5, 6]. These results have further underlined the important roles of SUMOs in all nuclear processes, revealing large numbers of transcriptional regulators, chromatin modifiers, and DDR factors as SUMO target proteins.

Whereas transcription factors and DDR factors are major groups of SUMO target proteins, little was known about how SUMO coordinates transcriptional regulation in response to DNA damage. We recently investigated the involvement of SUMO in the DDR at a proteome wide level, using the latest proteomics methods and tools available to quantitatively study changes in protein SUMOylation in response to cellular treatment with the DNA damaging agent methyl methanesulfonate (MMS) [7]. Through usage of an unbiased and system-wide approach, we identified subsets of 20 and 33 proteins being upregulated and downregulated in SUMOylation, respectively, in response to DNA damage. Furthermore, we quantitatively mapped 755 SUMOylation sites of which 362 were dynamic in response to MMS. Strikingly, we observed a cluster of chromatin modifiers, transcription factors and DNA repair factors to be differentially SUMOylated, providing new insight in how SUMO coordinates transcription in reaction to DNA damage.

**Figure 1:**
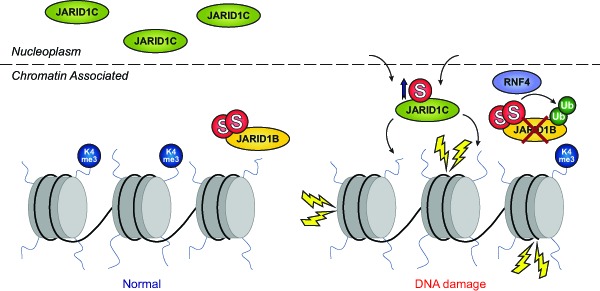
SUMO regulates KDM5B/JARID1B and KDM5C/JARID1C in response to DNA damage Subsets of chromatin remodelers, DNA repair factors and transcription factors are differentially SUMOylated in response to MMS treatment. Among these are the histone demethylases JARID1B and JARID1C. JARID1B normally resides at the chromatin and demethylates H3K4me3 preventing transcription of a subset of genes related to cell cycle progression and the DDR. Upon MMS treatment, JARID1B is rapidly degraded with involvement of RNF4 and the ubiquitin-protease system. Conversely, JARID1C normally resides in the nucleoplasm, and is recruited to the chromatin in response to MMS where it globally demethylates H3K4me3 and thereby downregulates transcription levels.

Interestingly, cells entered a repressed transcriptional state in response to MMS, as monitored at the level of histone modifications, and differential SUMOylation of various chromatin remodelers could account for the observed effect. Mechanistically, we found that two related histone demethylases, KDM5B/JARID1B and KDM5C/JARID1C, were differentially regulated by SUMOylation in response to MMS. JARID1B was degraded in a SUMO-dependent manner, regulated by the SUMO-targeted ubiquitin ligase RNF4, and JARID1C was SUMOylated and recruited to the chromatin in order to globally demethylate histone H3 lysine-4, causing transcriptional repression (Figure 1). Thus, JARID1C acts as a chromatin modifier which represses transcription as part of the DDR [7]. In contrast, the degradation of JARID1B led to transcriptional activation of selected genes, including *JUN* and *MCL1*, which are normally repressed by JARID1B.

Additionally, SUMO orchestrated other chromatin remodelers in response to DNA damage in order to induce overall transcriptional repression, including MBD1, SETDB1, P300, and CBP. Thus, not only can SUMO control transcription, chromatin organization, and the DDR separately, but SUMO is also able to integrate these pivotal processes in a coordinated manner, highlighting the role of SUMO as a critical integrator of different nuclear processes to cope with DNA damage.
